# Correction: Graphene oxide-iron oxide and reduced graphene oxide-iron oxide hybrid materials for the removal of organic and inorganic pollutants

**DOI:** 10.1039/d2ra90103j

**Published:** 2022-10-11

**Authors:** Xin Yang, Changlun Chen, Jiaxing Li, Guixia Zhao, Xuemei Ren, Xiangke Wang

**Affiliations:** Key Laboratory of Novel Thin Film Solar Cells, Institute of Plasma Physics, Chinese Academy of Sciences P. O. Box 1126 Hefei 230031 P. R. China clchen@ipp.ac.cn xkwang@ipp.ac.cn +86 5515591310 +86 551 5592788

## Abstract

Correction for ‘Graphene oxide-iron oxide and reduced graphene oxide-iron oxide hybrid materials for the removal of organic and inorganic pollutants’ by Xin Yang *et al.*, *RSC Adv.*, 2012, **2**, 8821–8826, https://doi.org/10.1039/C2RA20885G.

The authors regret that there is an error in the inset of Fig. 2. We made a calculation mistake by inadvertently plotting emu rather than emu g^−1^ as the *Y*-axis in the original article. The corrected inset for Fig. 2 is provided below.

**Fig. 2:**
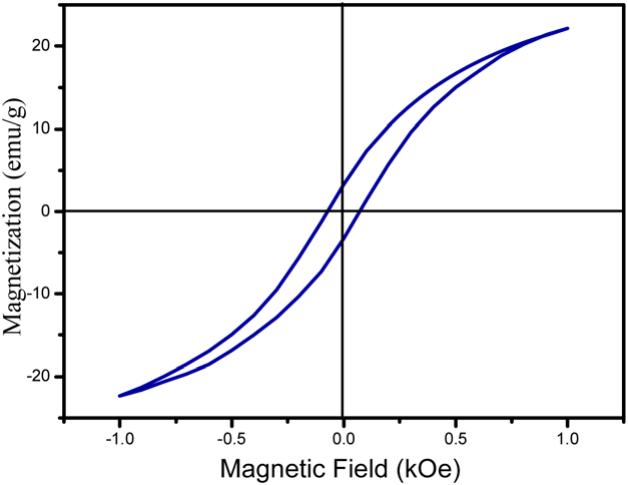
(inset) Magnetization curve at room temperature of the GO-iron oxides.

In addition, we would like to update section ‘2.2 Characterization’ to include the following information.

“The magnetic measurement was performed on an MPMS 3 (Quantum-Design) at room temperature and the magnetic moment was measured in the magnetic field range of −20.0 to +20.0 kOe.”

The authors confirm that this correction does not affect the discussion and conclusions of the original article. The authors would like to apologize for any inconvenience caused.

An independent expert reviewed the raw data provided by the authors and concluded that it was consistent with the corrected figure and does not change the discussions or conclusions presented in the article.

The Royal Society of Chemistry apologises for these errors and any consequent inconvenience to authors and readers.

## Supplementary Material

